# Comparison of One-Year Postoperative Outcomes Between Phacoemulsification With iStent Inject W and Phacoemulsification With Microhook Trabeculotomy: Real-World Data and Propensity Score-Matched Analysis

**DOI:** 10.7759/cureus.80979

**Published:** 2025-03-22

**Authors:** Azusa Yamagishi, Yuta Kitamura, Takayuki Baba

**Affiliations:** 1 Ophthalmology, Chiba University, Chiba, JPN

**Keywords:** intraocular pressure (iop), istent inject w, minimally invasive glaucoma surgery (migs), propensity score matching (psm), tanito microhook trabeculotomy (tmh)

## Abstract

Purpose

This study aimed to compare the one-year postoperative outcomes of cataract surgery combined with the iStent inject W trabecular micro-bypass stent (iStent IW) implantation and cataract surgery combined with microhook trabeculotomy (μLOT) in a real-world clinical setting, before and after propensity score matching (PSM).

Methods

This retrospective comparative chart review included 41 eyes in the iStent IW group and 72 in the μLOT group. The primary outcomes assessed were postoperative intraocular pressure (IOP) and the number of glaucoma medications. Initially, we compared baseline characteristics and surgical outcomes between both groups before PSM. Then, PSM was performed to minimize baseline differences in glaucoma type, preoperative IOP, and the number of glaucoma medications, enabling a more accurate comparison of the IOP-lowering effects of both procedures.

Results

In the unadjusted analysis, the iStent IW group had a lower baseline IOP (14.5 ± 3.3 mmHg) than the μLOT group (19.3 ± 4.9 mmHg, p < 0.0001), as well as fewer medications (3.6 ± 1.3 vs. 4.8 ± 1.5, p < 0.0001). Furthermore, the iStent IW group primarily comprised patients with primary open-angle glaucoma and normal-tension glaucoma, whereas the μLOT group included patients with a wider variety of glaucoma types. At 12 months, IOP was comparable between the iStent IW group (12.7 ± 2.8 mmHg) and the μLOT group (14.0 ± 4.1 mmHg; p = 0.2883), although the number of medications remained lower in the iStent IW group (2.2 ± 1.5 vs. 3.3 ± 1.7; p = 0.0299). After PSM, no significant differences in IOP or medications were observed. Layered hyphema occurred more frequently in the μLOT group.

Conclusion

Both procedures had comparable IOP reduction; however, the μLOT group had more postoperative layered hyphema and required more medications in the unadjusted analysis.

## Introduction

Glaucoma is one of the leading causes of blindness worldwide. The most evidence-based approach for managing glaucoma involves reducing intraocular pressure (IOP), which can be achieved through medications, laser therapy, or surgical interventions [1,2]. Trabeculectomy has traditionally been the gold standard among surgical procedures due to its substantial IOP-lowering effect. However, the high rate of postoperative complications and postoperative management difficulties have been significant challenges [3].

In recent years, minimally invasive glaucoma surgery (MIGS) has gained significant attention as a safer and less invasive alternative to traditional surgical procedures. MIGS is defined by the American Glaucoma Society and the US Food and Drug Administration as surgical techniques that avoid extensive conjunctival or scleral incisions. These procedures are further classified into canal-based, suprachoroidal-based, and subconjunctival-based approaches [4]. Canal-based MIGS targets the trabecular meshwork to reduce resistance in the conventional aqueous outflow pathway. Among these techniques, the iStent inject W trabecular micro-bypass stent (iStent IW: Glaukos Corporation, San Clemente, CA) uses an implant-based approach. On the other hand, microhook trabeculotomy (μLOT) and the Kahook Dual Blade rely on incisional methods targeting the trabecular meshwork. Despite differences between these techniques, most MIGS procedures can be performed quickly under gonioscopic guidance. Several studies have compared canal-based MIGS regarding indications, surgical outcomes, and complications [5-10]. In Japan, the use of iStent IW is regulated by specific guidelines, restricting its application to patients with primary open-angle glaucoma (POAG), normal-tension glaucoma (NTG), or pseudoexfoliation glaucoma (PXG) who have IOP below 25 mmHg while receiving glaucoma medication. In contrast, μLOT and Kahook Dual Blade are not governed by such guidelines, leading to broader indications compared to the iStent IW. However, iStent IW is associated with fewer complications, which may make it more acceptable to patients undergoing surgery [9,10].

This study compared the outcomes of cataract surgery combined with iStent IW and cataract surgery combined with μLOT in real-world clinical settings. To comprehensively evaluate these procedures, treatment outcomes both before and after propensity score matching (PSM) were analyzed. We compared results in unadjusted PSM to evaluate the baseline characteristics of patients who underwent each procedure and analyzed the surgical outcomes within this context. Additionally, by performing PSM to minimize baseline differences, we aimed to more accurately compare the IOP-lowering effects of both procedures.

## Materials and methods

Participants

This retrospective comparative chart review was based on chart reviews. Patients who underwent cataract surgery combined with iStent IW implantation or μLOT at Chiba University Hospital (Chiba, Japan) between May 2022 and October 2023 were included. The indication for iStent IW followed Japanese guidelines, targeting patients with POAG, NTG, or PXG with an IOP below 25 mmHg under treatment with glaucoma eye drops.

Exclusion Criteria and Eye Selection

The following were excluded from the study: (i) patients with a history of prior glaucoma surgery and (ii) patients with severe corneal diseases or retinal disorders that affected their vision (such disorders were not observed in the patient population). For patients who underwent surgery on both eyes, only the right eye was included in the analysis to avoid inter-eye correlation bias. This approach ensured that each patient contributed only one data point, preventing potential statistical dependence between the two eyes from the same patient. In total, 41 eyes were assigned to the iStent IW group, and 72 were assigned to the μLOT group.

Ethical Considerations

This study adhered to the principles of the Declaration of Helsinki and was approved by the Institutional Review Board (IRB) of Chiba University Hospital (IRB No. HK202405-13, with a revised protocol issued on November 25, 2024). As this was a retrospective study, specific informed consent for participation was not required, although the participants were informed of the study through posted protocols within the institution.

Measurements

Data collected from patient chart reviews included patient age, sex, glaucoma type, surgical history, preoperative best-corrected visual acuity (BCVA), preoperative visual field mean deviation (MD) (Central 30-2 Program, Humphrey Visual Field Analyzer, Carl Zeiss Meditec, Inc., Dublin, CA), and preoperative corneal endothelial cell density (EM-3000, TOMEY Corporation, Nagoya, Japan). Baseline IOP and the number of glaucoma medications were recorded, along with measurements at one, three, six, and 12 months postoperatively. IOP was measured using a Goldmann applanation tonometer. Visual field testing results within one year before surgery were included. Transient IOP elevation was defined as IOP exceeding 30 mmHg. Decimal BCVA values were converted to the logarithm of the minimal angle of resolution for analysis.

The addition of glaucoma medications was determined at the surgeon’s discretion, without strict criteria for restarting medications.

Surgical procedures

A 2.2 mm temporal corneal incision was first created in both procedures, followed by phacoemulsification and intraocular lens implantation. Viscoelastic material (Shellgan, Santen Pharmaceutical Co., Ltd., Osaka, Japan) was then injected into the anterior chamber, and the angle was visualized using a Hill gonio prism lens (Ocular Instruments Inc, Bellevue, WA). In the μLOT group, the nasal trabecular meshwork was incised over 90-120° using the Tanito microhooks (M-2215S, 2215R, and 2215L; Inami & Co., Ltd., Tokyo, Japan), with additional temporal incisions of 90-120° performed at the surgeon's discretion. In the iStent IW group, two iStent IW devices were implanted into the nasal trabecular meshwork. After trabeculotomy or stent implantation, viscoelastic material was aspirated, and the surgery was completed. Although all procedures followed standardized surgical protocols, some variability in technique among surgeons may have occurred. However, all surgeries were performed by experienced glaucoma specialists, and any potential variations are expected to have minimal impact on the overall outcomes.

Postoperatively, patients in the iStent IW group were prescribed topical 0.5% levofloxacin, 0.1% betamethasone, diclofenac sodium (four times daily), and tropicamide (once daily) for one to three months. In the μLOT group, topical 0.5% levofloxacin, 0.1% betamethasone, 2% pilocarpine (four times daily), and bromfenac (twice daily) were prescribed over the same period.

Statistical analyses

The primary outcomes were postoperative IOP and the number of glaucoma medications. The secondary outcomes included postoperative complications and survival rates analyzed using Kaplan-Meier survival curves. All data were de-identified and analyzed using JMP Pro 17 (SAS Institute Inc., Cary, NC). Pre- and postoperative data were compared using the Wilcoxon signed-rank test. Continuous variables between groups were compared using the Mann-Whitney U test, and categorical variables were compared using Fisher’s exact test. The success rates of IOP control were evaluated using Kaplan-Meier survival curves, with comparisons made using the log-rank test. Failure was defined as IOP exceeding 15 mmHg, surpassing preoperative levels, need for additional glaucoma surgery, or loss of light perception three months or later.

To minimize confounding factors, PSM was used to adjust for baseline differences between the two treatment groups, specifically in glaucoma type, preoperative IOP, and the number of glaucoma medications. This method helps create more comparable groups by minimizing selection bias, ensuring that observed differences in outcomes are due to treatment rather than pre-existing differences in patient characteristics. Matching was conducted with 1:1 nearest neighbor matching using a caliper width of 0.25. This resulted in the matching of 22 eyes from the iStent IW group and 22 eyes from the μLOT group.

Statistical significance was set at p < 0.05 and considered statistically significant. Continuous variables were expressed as mean ± standard deviation, whereas categorical variables were presented as numbers and percentages.

## Results

Table 1 summarizes the participant demographic details before PSM. There were no significant differences between the two groups for age, sex, preoperative BCVA, or preoperative corneal endothelial cell density. However, preoperative MD was significantly higher in the iStent IW group. In contrast, preoperative IOP and the number of glaucoma medications were significantly lower in the iStent IW group than in the μLOT group. Regarding glaucoma type, the iStent IW group primarily included patients with POAG and NTG, whereas the μLOT group included a wider range of glaucoma types, such as primary angle-closure glaucoma (PACG), PXG, and uveitis-induced glaucoma.

**Table 1:** Demographic and clinical characteristics before propensity score matching Data are presented as mean ± SD (range) or number (%). Statistical significance was set at p<0.05. ^a^: Mann–Whitney U test, ^b^: Fisher’s exact test iStent IW: iStent inject W trabecular micro-bypass stent; μLOT: Microhook trabeculotomy; IOP: Intraocular pressure; BCVA: best-corrected visual acuity; HFA: Humphrey field analyzer; MD: Mean deviation; CECD: corneal endothelial cell density; POAG: primary open-angle glaucoma; NTG: normal tension glaucoma; PACG, primary angle-closure glaucoma; PXG: pseudoexfoliation glaucoma; Uveitis; uveitis-induced glaucoma; Steroid: steroid-induced glaucoma

Items	iStent IW	μLOT	p-value
N (eyes)	41	72	
Age	74.0 ± 6.9 (59– 87)	75.8 ± 7.1 (54– 90)	0.1924^a^
Sex (female/male)	25/16	51/21	0.3037^b^
IOP (mmHg)	14.5 ± 3.3 (10– 20)	19.3 ± 4.9 (11– 40)	<0.0001^a^
Number of medications	3.6 ± 1.3 (1–5)	4.8 ± 1.5 (2– 7)	0.0002^a^
BCVA (LogMAR)	0.37 ± 0.35 (0.05– 1.7)	0.41 ± 0.43 (-0.1–2.3)	0.7248^a^
HFA MD (dB)	-11.2 ± 6.2 (-27.9– -3.2)	-14.3 ± 7.0 (-27.6–0.43)	0.0216^a^
CECD (cells/mm2)	2450.0 ± 318.3 (1251– 2932)	2400.6 ± 390.9 (1147–3193)	0.3906^a^
Glaucoma type, eyes (%)			
POAG	19 (46.3%)	27 (37.5%)	0.4270^b^
NTG	20 (48.8%)	2 (2.8%)	<0.0001^b^
PACG	0	9 (12.5%)	0.0251^b^
PXG	2 (4.9%)	21 (29.2%)	0.0015^b^
PACG + PXG	0	1 (1.4%)	1.0000^b^
Uveitis	0	7 (9.7%)	0.0471^b^
Steroid	0	1 (1.4%)	1.0000^b^
Uveitis + Steroid	0	3 (4.2%)	0.5524^b^

Table 2 summarizes postoperative complications before PSM. Layered hyphema occurred significantly more frequently in the μLOT group than in the iStent IW group. In the μLOT group, three cases exhibited multiple complications: one had layered hyphema and transient IOP elevation, another had layered hyphema and corneal edema, and the third had rheumatoid arthritis-associated uveitis and transient IOP elevation. Herpes iritis and RA-associated uveitis occurred in eyes with a history of uveitis, suggesting these conditions may have been triggered by postoperative inflammation. Central retinal vein occlusion developed two weeks after surgery, making a direct causal relationship with the procedure unlikely and suggesting it was more likely an incidental finding.

**Table 2:** Postoperative complications and adverse events before propensity score matching Data are presented as number (%). Statistical significance was set at p<0.05. ^b^: Fisher’s exact test iStent IW: iStent inject W trabecular micro-bypass stent; μLOT: Microhook trabeculotomy; IOP: Intraocular pressure; RA: rheumatoid arthritis

Items	iStent IW	μLOT	p-value
Layered hyphema	1 (2.4%)	15 (20.8%)	0.0095^b^
Transient IOP elevation (mmHg)	1 (2.4%)	7 (9.7%)	0.2546^b^
Corneal edema	1 (2.4%)	5 (6.9%)	0.4145^b^
Corneal erosions	0	1 (1.4%)	1.0000^b^
Retinal central macular edema	0	1 (1.4%)	1.0000^b^
Herpes iritis	0	1 (1.4%)	1.0000^b^
RA-associated uveitis	0	1 (1.4%)	1.0000^b^
Central retinal vein occlusion	0	1 (1.4%)	1.0000^b^

**Figure 1:**
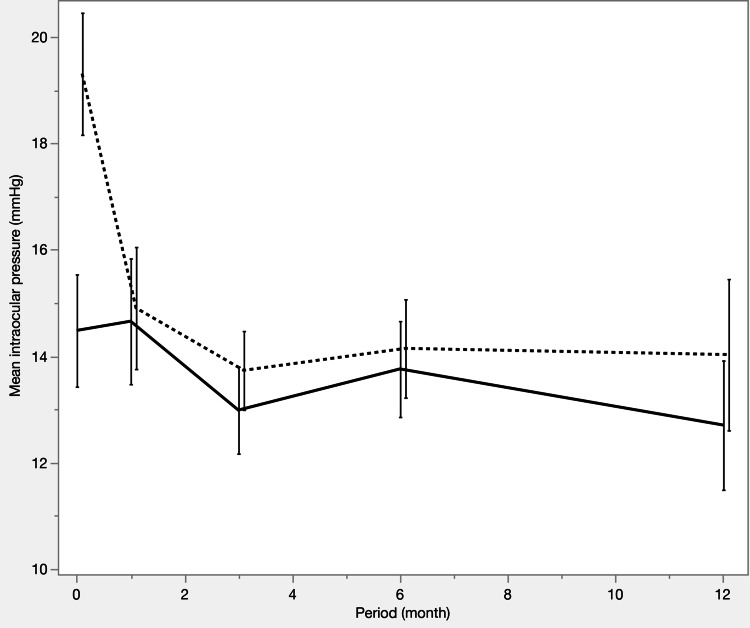
Intraocular pressure (IOP) changes before propensity score matching Mean IOP decreased significantly in both groups from baseline. Preoperative IOP was lower in the iStent IW group (p < 0.0001), while postoperative IOP was comparable between groups (p = 0.1813 at 12 months). Solid line: iStent IW group, dashed line: μLOT group

**Figure 2:**
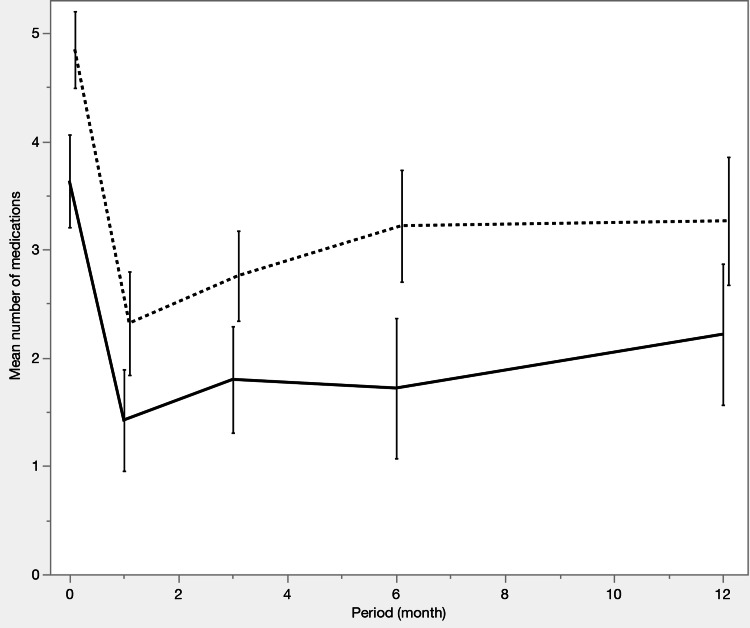
Changes in the number of medications before propensity score matching The number of glaucoma medications decreased significantly in both groups from baseline. The iStent IW group required fewer medications than the μLOT group throughout the study period (p < 0.0001 at baseline; p = 0.0202 at 12 months). Solid line: iStent IW group, dashed line: μLOT group

**Figure 3:**
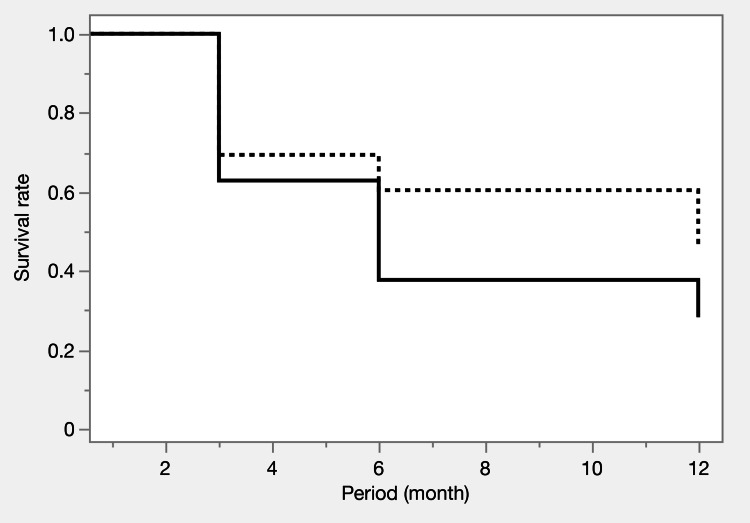
Kaplan-Meier survival curves before propensity score matching Survival curves for IOP control at 12 months show rates of 28.3% (iStent IW) and 46.0% (μLOT), with no significant difference (p = 0.1283). Failure criteria included elevated IOP, additional surgery, or vision loss. Solid line: iStent IW group, dashed line: μLOT group

Table 3 and Figures 1-3 summarize the IOP trends, number of medications, and survival curves. Both groups had a significant reduction in IOP and the number of medications from preoperative to postoperative levels. At 12 months postoperatively, IOP was comparable between the two groups (12.7 ± 2.8 vs 14.0 ± 4.1 mmHg; p = 0.2883), although the number of medications was significantly lower in the iStent IW group than in the μLOT group (2.2 ± 1.5 vs. 3.3 ± 1.7; p = 0.0299). The survival curve analysis showed no significant differences between the two groups.

**Table 3:** One-year postoperative outcomes before propensity score matching Data are presented as mean ± SD (range). Statistical significance was set at p<0.05. *: Wilcoxon signed-rank test (comparison between preoperative and postoperative values) **: Mann–Whitney U test (comparison between the groups at each visit) iStent IW: iStent inject W trabecular micro-bypass stent; μLOT: Microhook trabeculotomy; IOP: intraocular pressure

Items	iStent IW	p.value*	μLOT	p.value*	p.value**
Baseline					
IOP (mmHg)	14.5±3.3 (10– 20)		19.3±4.9 (11– 40)		<0.0001
Number of medications	3.6±1.3 (1–5)		4.8±1.5 (2–7)		0.0002
N (eyes)	41		72		
1 month after operation					
IOP (mmHg)	14.7±3.7(8–28)	0.6290	14.9±4.9 (9–38)	<0.0001	0.8834
Number of medications	1.4±1.5 (0–5)	<0.0001	2.3±2.0 (0–7)	<0.0001	0.0283
N (eyes)	40		72		
3 months after operation					
IOP (mmHg)	13.0±2.3(10–18)	0.0006	13.7±2.9(8–24)	<0.0001	0.2361
Number of medications	1.8±1.4(0–5)	<0.0001	2.8±1.6(0–7)	<0.0001	0.0056
N (eyes)	35		62		
6 months after operation					
IOP (mmHg)	13.8±2.2 (10–19)	0.2107	14.1±2.9 (9–21)	<0.0001	0.6410
Number of medications	1.7±1.6 (0–5)	<0.0001	3.2±1.6 (0–7)	<0.0001	0.0010
N (eyes)	25		41		
12 months after operation					
IOP (mmHg)	12.7±2.8 (9–18)	0.0157	14.0±4.1 (8–26)	<0.0001	0.2883
Number of medications	2.2±1.5 (0–5)	0.0001	3.3±1.7 (0–7)	<0.0001	0.0299
N (eyes)	23		34		

Table 4 presents the demographic details of the participants after PSM. There were no significant differences between the two groups in all respects.

**Table 4:** Demographic and clinical characteristics after propensity score matching Data are presented as mean ± SD (range) or number (%). Statistical significance was set at p<0.05. ^a^: Mann–Whitney U test, ^b^: Fisher’s exact test iStent IW: iStent inject W trabecular micro-bypass stent; μLOT: Microhook trabeculotomy; IOP: Intraocular pressure; BCVA: best-corrected visual acuity; HFA: Humphrey field analyzer; MD: Mean deviation; CECD: corneal endothelial cell density; POAG: primary open-angle glaucoma; NTG: normal tension glaucoma; PXG: pseudoexfoliation glaucoma

Items	iStent IW	μLOT	p-value
N (eyes)	22	22	
Age	74.6 ± 6.8 (59–87)	77.0 ± 6.4 (63–89)	0.2690^a^
Sex (female/male)	13/9	16/6	0.5256^b^
IOP (mmHg)	16.6 ± 2.3 (10–20)	17.0 ± 2.2 (14–21)	0.6716^a^
Number of medications	3.8 ± 1.4 (1–5)	3.8 ± 1.1 (2–5)	0.8527^a^
BCVA (LogMAR)	0.39 ± 0.44 (0.05–1.7)	0.40 ± 0.27 (0.05–1.3)	0.2745^a^
HFA MD (dB)	-9.9 ± 4.9 (-19.3 to -3.2)	-13.3 ± 6.6 (-27.6– -3.3)	0.0580^a^
CECD (cells/mm^2^)	2469.7 ± 304.5 (1502–2932)	2430.6 ± 353.1 (1648–3193)	0.6144^a^
Glaucoma type, eyes (%)			
POAG	16 (72.7%)	18 (81.8%)	0.7205^b^
NTG	4 (18.9%)	2 (9.1%)	0.6640^b^
PXG	2 (9.1%)	2 (9.1%)	1.0000^b^

Table 5 summarizes the postoperative complications after PSM. Layered hyphema occurred significantly more frequently in the μLOT group than in the iStent IW group.

**Table 5:** Postoperative complications and adverse events after propensity score matching Data are presented as number (%). Statistical significance was set at p<0.05. ^b^: Fisher’s exact test iStent IW: iStent inject W trabecular micro-bypass stent; μLOT: Microhook trabeculotomy; IOP: intraocular pressure

Items	iStent IW	μLOT	p-value
Layered hyphema	1 (4.6%)	10 (45.5%)	0.0039^b^
Transient IOP elevation	1 (4.6%)	0	1.0000^b^
Corneal edema	1 (4.6%)	2 (9.1%)	1.0000^b^

**Figure 4:**
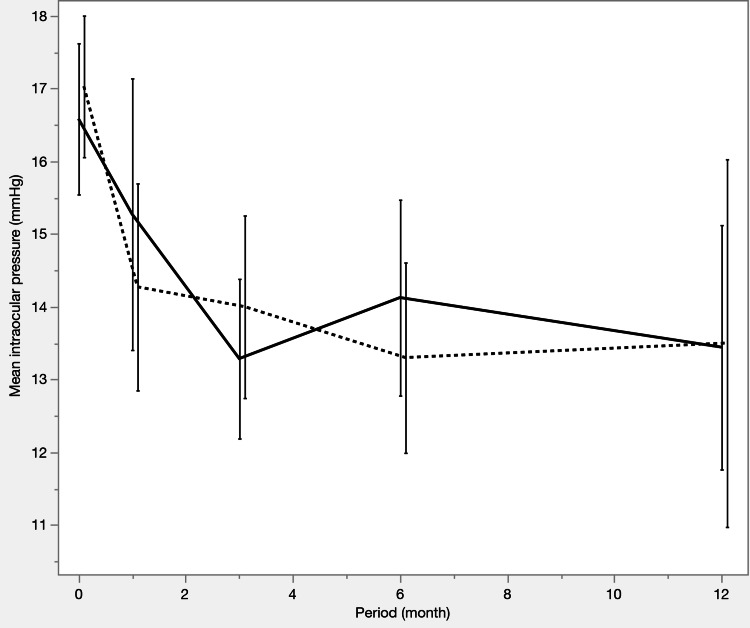
Intraocular pressure (IOP) changes after propensity score matching IOP decreased significantly in both groups from baseline, with similar levels observed at all postoperative visits, including 12 months (p = 1.0000). Solid line: iStent IW group, dashed line: μLOT group

**Figure 5:**
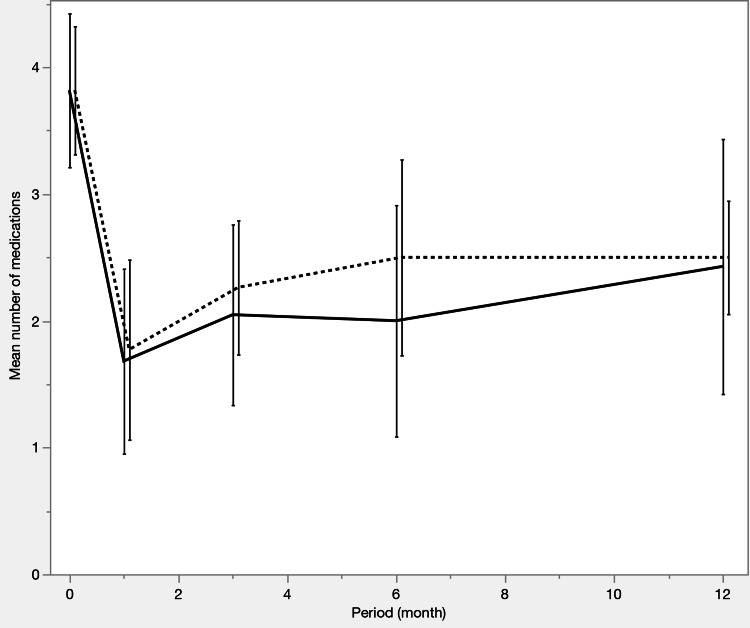
Changes in the number of medications after propensity score matching The number of glaucoma medications decreased significantly in both groups from baseline, except in the μLOT group at 12 months (p = 0.0625). No significant difference was observed between the two groups at 12 months (p = 1.0000). Solid line: iStent IW group, dashed line: μLOT group

**Figure 6:**
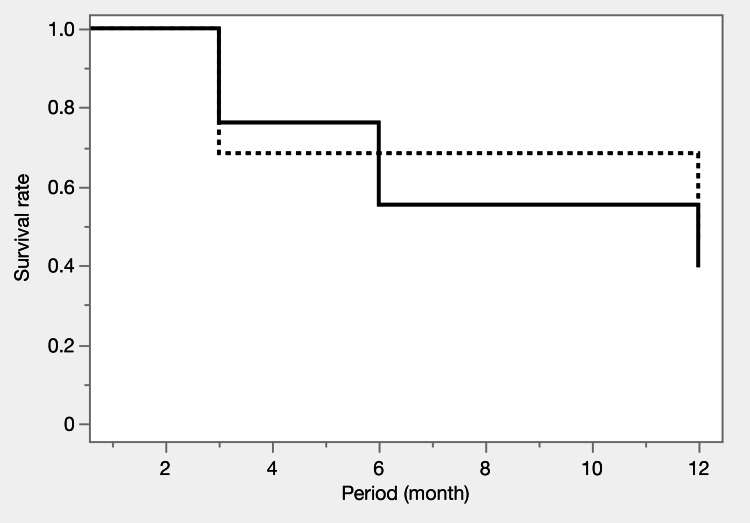
Kaplan-Meier survival curves after propensity score matching Survival curves for IOP control at 12 months show rates of 39.6% (iStent IW) and 45.6% (μLOT), with no significant difference (p = 0.8206). Failure criteria included elevated IOP, additional surgery, or vision loss. Solid line: iStent IW group, dashed line: μLOT group

Table 6 and Figures 4-6 summarize the IOP trends, number of medications, and survival curves after PSM. Both groups had a significant reduction in IOP and the number of medications postoperatively compared to that preoperatively, except for the μLOT group at 12 months (p = 0.0625), where the reduction in medication use was not statistically significant. No significant differences were observed between the two groups in these analyses.

**Table 6:** One-year postoperative outcomes after propensity score matching Data are presented as mean ± SD (range). Statistical significance was set at p<0.05. *: Wilcoxon signed-rank test (comparison between preoperative and postoperative values) **: Mann–Whitney U test (comparison between the groups at each visit) iStent IW: iStent inject W trabecular micro-bypass stent; μLOT: Microhook trabeculotomy; IOP: intraocular pressure

Items	iStent IW	p.value*	μLOT	p.value*	p.value**
Baseline					
IOP (mmHg)	16.6±2.3 (10–20)		17.0±2.2 (14–21)		0.6716
Number of medications	3.8±1.4 (1–5)		3.8±1.1 (2–5)		0.8430
N (eyes)	22		22		
1 month after operation					
IOP (mmHg)	15.3±4.2 (8–28)	0.0122	14.2±3.2 (10–22)	<0.0001	0.3074
Number of medications	1.7±1.6 (0–5)	<0.0001	1.7±1.6 (0–5)	<0.0001	0.8181
N (eyes)	22		22		
3 months after operation					
IOP (mmHg)	13.3±2.4 (9–16)	<0.0001	14.0±2.6 (10–20)	0.0003	0.5283
Number of medications	2.0±1.6 (0–5)	0.0006	2.3±1.1 (0–5)	0.0002	0.6101
N (eyes)	21		19		
6 months after operation					
IOP (mmHg)	14.1±2.5 (10–19)	0.0002	13.3±1.8 (10–16)	0.0078	0.3771
Number of medications	2.0±1.7 (0–5)	0.0024	2.5±1.1 (1–5)	0.0313	0.4474
N (eyes)	16		10		
12 months after operation					
IOP (mmHg)	13.4±2.9 (10–18)	0.0024	13.5±3.0 (10–19)	0.0078	1.0000
Number of medications	2.4±1.7 (0–5)	0.0137	2.5±0.5 (2–3)	0.0625	1.0000
N (eyes)	14		8		

Patient attrition and follow-up

As this study was a retrospective chart review, some patients with good postoperative outcomes were discharged from follow-up based on the judgment of their physician. The attrition rate was comparable between the two groups, and no cases were lost due to surgical complications. However, this potential selection bias due to loss to follow-up cannot be fully ruled out.

Medication adjustment criteria

There were no strict predefined criteria for postoperative glaucoma medication adjustments. However, in clinical practice, additional medications were often prescribed when IOP exceeded 15 mmHg. This variability in treatment approach may have influenced the number of medications recorded at different time points but reflects real-world clinical decision-making.

## Discussion

This study provides a novel perspective by combining real-world data with PSM to evaluate the outcomes of iStent IW and μLOT. In the unadjusted analysis, the μLOT group included patients with higher preoperative IOP, a greater number of medications, and more severe visual field defects compared to those in the iStent IW group. Additionally, the μLOT group had broader glaucoma subtypes, including patients with PACG, PXG, and SOAG, whereas the iStent IW group predominantly comprised patients with POAG and NTG. μLOT was applied to more severe cases and a broader range of disease types, likely because its use is not restricted by specific guidelines. In contrast, iStent IW was more frequently chosen for NTG cases because iStent IW tended to be preferred over μLOT for patients eligible for both procedures due to its lower risk of complications. Despite these baseline differences, postoperative IOP was comparable between the two groups, although the μLOT group required significantly more postoperative medications. This difference may have been attributed to the more complex baseline characteristics of the μLOT group. After applying PSM to adjust for the confounding factors of glaucoma type, preoperative IOP, and the number of glaucoma medications, no significant differences between the two groups concerning postoperative IOP or the number of medications were observed. However, in the μLOT group, the number of medications did not decrease significantly at 12 months (p = 0.0625). This may be due to the small sample size with limited statistical power for detecting significant differences. The incidence of layered hyphema remained significantly higher in the μLOT group regardless of PSM.

iStent IW and μLOT groups were compared in two retrospective studies [9,10]. In the study by Harano et al. [9], the μLOT group had higher preoperative IOP than the iStent IW group, whereas in the study by Onoe et al. [10], preoperative IOP and the number of medications were comparable in both groups. In both studies, the postoperative IOP and the number of medications were comparable in both groups, and layered hyphema remained higher in the μLOT groups (12.8%-26%) than in the iStent IW groups (1.8%-5%). A wider range of trabecular meshwork incisions may induce a higher rate of postoperative anterior chamber hemorrhage [11].

In the comparison before PSM in this study, postoperative IOP was comparable, and the number of postoperative medications was higher in the μLOT group. Although the results demonstrated that μLOT was effective in patients with high preoperative IOP or complicated disease types, the higher number of postoperative medications in the μLOT group contrasts with previous reports. This discrepancy may have resulted from the inclusion of patients with a broader range of disease types in the μLOT group in this study. Additionally, the results of the comparison after PSM were consistent with those of Onoe et al. [10]. By applying PSM to adjust for confounding factors such as glaucoma type, preoperative IOP, and the number of glaucoma medications, we were able to minimize the influence of baseline differences and provide a more balanced comparison between iStent IW and μLOT. This analysis demonstrated that both procedures had similar efficacy in reducing IOP. The similarity in postoperative IOP between the two groups, both in the unadjusted and PSM analyses, aligns with the broader literature on canal-based MIGS. Larger incision widths in the trabecular meshwork may lower outflow resistance [12]; however, the clinical differences in outcomes remain minimal. For example, comparisons between one-quadrant and two-quadrant μLOT did not have significant differences in postoperative outcomes [11]. Similarly, studies comparing various canal-based MIGS have reported comparable results [5-10]. This may be explained by the physiological mechanism of canal-based MIGS: the reduction in IOP diminishes as IOP approaches episcleral venous pressure, regardless of the extent of trabecular meshwork intervention. This inherent limitation is likely responsible for the consistent findings across different procedures, including the results of this study.

This study has several limitations. A major limitation of the study is its retrospective nature, which may have introduced selection bias. The criteria for adding glaucoma medications were determined at the discretion of the attending physicians, which may have introduced bias. Despite using PSM to reduce confounding, the sample size was small, with 14 cases in the iStent IW group and eight cases in the μLOT group at 12 months. The limited sample size likely contributed to the lack of statistical significance in the μLOT group at 12 months, as power analysis using a t-test approximation indicated only 44.4% power for detecting a significant reduction in medication use. Future prospective randomized controlled trials with larger sample sizes are warranted to confirm these findings. Future prospective randomized controlled trials with larger sample sizes are warranted to confirm these findings.

## Conclusions

μLOT was performed in patients with more severe and broader glaucoma types compared to those who underwent iStent IW, and postoperative IOP results were comparable. After PSM, no significant differences were observed between the procedures. Layered hyphema consistently occurred more frequently in the μLOT group regardless of PSM. Therefore, although μLOT was effective for patients with complex types of glaucoma, iStent IW may be more beneficial for patients with both iStent IW and μLOT indicated due to its low complication rate.
